# Identification of immunotherapy and chemotherapy-related molecular subtypes in colon cancer by integrated multi-omics data analysis

**DOI:** 10.3389/fimmu.2023.1142609

**Published:** 2023-03-20

**Authors:** Jie Zhu, Weikaixin Kong, Liting Huang, Suzhen Bi, Xuelong Jiao, Sujie Zhu

**Affiliations:** ^1^ Key Laboratory of Birth Regulation and Control Technology of National Health Commission of China, Shandong Provincial Maternal and Child Health Care Hospital Affiliated to Qingdao University, Jinan, Shandong, China; ^2^ Institute of Translational Medicine, The Affiliated Hospital of Qingdao University, College of Medicine, Qingdao University, Qingdao, China; ^3^ Institute for Molecular Medicine Finland (FIMM), HiLIFE, University of Helsinki, Helsinki, Finland; ^4^ Department of Molecular and Cellular Pharmacology, School of Pharmaceutical Sciences, Peking University Health Science Center, Beijing, China; ^5^ Gastrointestinal Surgery Department, The Affiliated Hospital of Qingdao University, College of Medicine, Qingdao University, Qingdao, China

**Keywords:** bioinformatics, colon cancer, machine learning, immune therapy, multiple omics

## Abstract

**Background:**

Colon cancer is a highly heterogeneous disease, and identifying molecular subtypes can provide insights into deregulated pathways within tumor subsets, which may lead to personalized treatment options. However, most prognostic models are based on single-pathway genes.

**Methods:**

In this study, we aimed to identify three clinically relevant subtypes of colon cancer based on multiple signaling pathways-related genes. Integrative multi-omics analysis was used to explain the biological processes contributing to colon cancer aggressiveness, recurrence, and progression. Machine learning methods were employed to identify the subtypes and provide medication guidance for distinct subtypes using the L1000 platform. We developed a robust prognostic model (MKPC score) based on gene pairs and validated it in one internal test set and three external test sets. Risk-related genes were extracted and verified by qPCR.

**Results:**

Three clinically relevant subtypes of colon cancer were identified based on multiple signaling pathways-related genes, which had significantly different survival state (Log-Rank test, p<0.05). Integrative multi-omics analysis revealed biological processes contributing to colon cancer aggressiveness, recurrence, and progression. The developed MKPC score, based on gene pairs, was robust in predicting prognosis state (Log-Rank test, p<0.05), and risk-related genes were successfully verified by qPCR (t test, p<0.05). An easy-to-use web tool was created for risk scoring and therapy stratification in colon cancer patients, and the practical nomogram can be extended to other cancer types.

**Conclusion:**

In conclusion, our study identified three clinically relevant subtypes of colon cancer and developed a robust prognostic model based on gene pairs. The developed web tool is a valuable resource for researchers and clinicians in risk scoring and therapy stratification in colon cancer patients, and the practical nomogram can be extended to other cancer types.

## 1 Introduction

Colon cancer is a disease with extensive interpatient heterogeneity, both molecularly and histopathologically, which cannot be resolved by current clinical methods. Despite a continuous refinement to the UICC tumor, node, metastasis (TNM) staging system to measure disease extent and define prognosis, disease outcome still varies considerably even among patients with the same tumor stage. Therefore, new factors that can more precisely stratify patients into different risk categories are clearly warranted (1, 2).

In this age of advanced molecular-profiling technologies, cancer molecular subtype discovery has become one of the more common exercises utilizing transcriptomic data on human tumors. Molecular subtypes can deepen our understanding of cancer as a collection of diseases rather than a single disease. Molecular subtypes can provide insights into the pathways that appear deregulated within tumor subsets, which may suggest therapeutic opportunities, as well as being indicative of which pathways, as characterized in the experimental setting, would appear particularly relevant in the human disease setting (3).

As a highly heterogeneous disease, colon cancer involves DNA repair defects (4, 5), DNA methylation (6, 7), chromosome instability (8), and other molecular pathogeneses during disease development. Biomarkers have been used as common tools for disease detection and prognosis management in colon cancer patients. Therefore, the determination of molecular changes in colon cancer patients has become a hotspot in colon cancer research (9).

Recent attempts to resolve colorectal cancer (CRC) heterogeneity and improve prognosis include molecular subclassification and characterization based on transcriptional profiling (10, 11). The consensus molecular subtype (CMS) classification stratifies CRC into four subtypes CMS 1–4, each with distinct biological and histopathological features. Colorectal cancer is a molecularly heterogeneous disease. Responses to genotoxic chemotherapy in the adjuvant or palliative setting vary greatly between patients, and colorectal cancer cells often resist chemotherapy by evading apoptosis (12, 13). The development of cancer was related to multiple signaling pathways, including the cell cycle, immunity, aging, metabolism, autophagy, and so on. Until recently, most constructed prognostic models were based on single-pathway genes. Herein, we identified three clinically relevant subtypes of colon cancer based on multiple prognostic cancer signaling pathway-related genes. Integrative multi-omics analysis is used to explain the biological processes contributing to colon cancer aggressiveness, recurrence, and progression. We developed a classifier to identify the subtypes of patients and predicted medication guidance for each subtypes using the L1000 platform (14). Finally, we established a prognostic model system based on gene pairs using expression data and further validated it in one internal test set and three external test sets.

## 2 Methods

### 2.1 Colon cancer dataset source and preprocessing

The workflow of our study is shown in **Supplementary Figure S1A**. Public gene-expression data and full clinical annotation were obtained from the Gene-Expression Omnibus (GEO) and The Cancer Genome Atlas (TCGA) databases. Patients without survival information were removed. In total, three colon cancer cohorts (TCGA-COAD, GSE39582, and GSE38832; the data information is in **Supplementary Table S1**) were gathered in this study for further analysis. TCGA-COAD was downloaded from the Genomic Data Commons (GDC, https://portal.gdc.cancer.gov/). The somatic mutation data were acquired from TCGA database. The genomic instability (GI) and somatic copy-number alterations (SCNAs) of TCGA were downloaded from a previous study (15) (**Supplementary Table S3**).

### 2.2 Unsupervised clustering for 66 prognostic genes

Firstly, we searched the articles using the keywords “colon cancer” and “prognosis” to obtain the genes related to the prognosis of colon cancer and then identified 66 prognostic genes using univariate Cox regression. Unsupervised clustering was then used to identify three subtypes of colon cancer patients based on the expression of these 66 prognostic genes. We used the ConsensuClusterPlus package to perform the above steps, and 1,000 repetitions were conducted to guarantee the stability of clustering. Partitioning around medoid (PAM) method and Euclidean distance were used to quantify the similarity of gene expression profiles between the patients, and the area under the curves of the cumulative distribution function (CDF) was used to find the optimal number k of clusters.

### 2.3 PD1/CTLA4 response prediction

To predict the immunotherapy response of patients with distinct subtypes of breast cancer, we downloaded the immunotherapy prediction information from the TCIA database (https://tcia.at/home), which provides results of comprehensive immunogenomic analyses of next-generation sequencing data (NGS) for 20 solid cancers from TCGA and other data sources. The immunophenoscore (IPS) can be used to predict the response to the immunotherapy agents PD1 and CTLA4 (**Supplementary Table S6**).

### 2.4 Gene-set variation analysis and functional annotation

To investigate the differences in biological processes between three subtypes of colon cancer, we performed gene-set variation analysis (GSVA) using “GSVA” R packages. GSVA, a non-parametric and unsupervised method, is commonly used to estimate variation in pathway and biological process activity in expression data. The gene sets of “c2.cp.kegg.v6.2.symbols” were downloaded from the MsigDB database for running GSVA analysis.

### 2.5 Estimation of TME cell infiltration

We used the single-sample gene-set enrichment analysis (ssGSEA) algorithm to quantify the relative abundance of each cell infiltration in colon cancer tumor microenvironment (TME). The gene set for marking each TME infiltration immune cell type was obtained from the study of Charoentong (15), which stored various human immune cell subtypes including activated CD8 T cell, activated dendritic cell, macrophage, nature killer T cell, regulatory T cell, and so on. The enrichment scores calculated by ssGSEA analysis were utilized to represent the relative abundance of each TME infiltration cell in each sample. We used the limma, GSEABase, ggpubr, and reshape2 packages in R in this step.

### 2.6 Feature selection of each subtype of colon cancer compared with normal colon and drug analysis

To identify the marker genes for each subtype of colon cancer patients, the empirical Bayesian approach of the limma R package was applied to determine differentially expressed genes (DEGs) between cluster A/B/C and normal colon, respectively. The criteria for determining DEGs were set at an adjusted *p*-value of< 0.01. At the same time, weighted gene co-expression network analysis (WGCNA) was used to identify the related genes of subtypes of cancer (RG). Next, the protein–protein interaction (PPI) network was further used to screen the hub genes of the intersection of DEGs and RGs using String and Cytoscape software. Maximal Clique Centrality (MCC) was used to screen hub genes (the most connected genes). After obtaining the most connected genes, they were used to perform L1000 to screen drugs for each subtype. The final drug screening criteria of L1000 were set as score< −0.90.

### 2.7 Prognostic model building

Firstly, TCGA dataset was divided into a training set and an internal test set. Among the 66 prognostic genes, we paired these genes to address the batch effect in the training set. If the expression of gene A > the expression of gene B, then the feature “A|B” is marked as 1, otherwise, it is marked as 0, as shown in Eq. (1).


(1) $$\text{Feature}:\;"\text{Gene A}│\text{Gene B}"=\{\begin{array}{c} 1,\;\text{Expression}\left(A\right)>\text{Expression}\left(B\right)\\ 0,\;\text{Expression}\left(A\right)\leq \text{Expression}\left(B\right) \end{array}$$

In addition, if the expression level of gene A in all the samples is higher than the expression of gene B, then Gene A|Gene B is marked as 1 in all the samples. Such features do not contain classification information, and therefore we delete the gene pairs whose frequency of the “1” label in the training set is less than 0.2 or greater than 0.8. Next, univariate Cox regression and LASSO regression were used to reduce the number of these paired gene features in the training set. Finally, multivariate Cox regression was used to construct the multiple key cancer processes related to gene-pair score (MKPC). The “glmnet,” “survival,” and “survminer” packages in R were used in the above analysis process.

### 2.8 Classifier constructing

To make genotyping available to other researchers, we compared two methods based on the expression of the 66 prognostic genes. (1) We used the center points of the three subtypes in the training set (TCGA-COAD) (partitioning around medoid clustering method) to classify the new samples. The label of each new sample depends on the nearest center point of the sample. (2) We use the training set (TCGA-COAD) to build a multi-layer perceptron model (MLP) to label new samples. This MLP model contains three layers, which have 16, 64, and 64 neurons, respectively. We first used 10-fold cross-validation (CV) on the training set to perform a grid search to find the optimal model parameters for accuracy. The parameters in grid search are: “activation” is one of “identity,” “logistic,” “tanh,” or “relu”; “alpha” is one of 0.00001, 0.0001, 0.001, 0.01, and 0.1; “solver” is one of “lbfgs,” “sgd,” or “adam.” The MLP model with the highest accuracy in CV is used to predict the test set.


Accuracy=N(patients predicted correctlty)/N(all patients)

The above methods (1) and (2) were used to make predictions in the test set. We conducted a survival analysis (log-rank test) based on the prediction results. The method with a smaller *p*-value was used to build a website (https://sujiezhulab.shinyapps.io/coad/) by using the shiny package in R, which can be used by other researchers.

### 2.9 Prognostic model validation

To investigate the prognostic performance of the MKPC score, we tested it in four colon cancer patient cohorts (three external sets and one internal set). We then calculated the area under the curve (AUC) of the receiver operating characteristic (ROC) for overall survival (OS) time prediction. The models were evealuated using their 1-, 3-, and 5-year AUC values.

### 2.10 Tumor mutation burden analysis

The mutation data were downloaded from the GDC Data Portal (https://portal.gdc.cancer.gov/) and intersected with the samples with expression data. After that, we obtained 397 patient samples containing both expression data and mutation data. For these patients, we used the “maftools” package in R to plot a waterfall chart and mutation gene cloud chart, obtain differential mutated genes (DMGs) between different subtypes of colon cancer, and calculate the tumor mutation burden (TMB) value by finding out the number of gene mutations per million bases. The Wilcoxon test was used to compare the TMB values of the MKPC high- and low-risk groups.

## 3 Results

### 3.1 Construction of three molecular subgroups of colon cancer using prognostic genes

Cancer is a systemic, complex disease related to abnormalities in multiple signaling pathways. In this study, we searched PubMed for studies related to the prognosis of colon cancer and obtained 183 genes from different signaling pathways (**Supplementary Table S2**). Next, we identified 66 prognostic genes (*p<* 0.05; **Supplementary Table S3**) using univariate Cox regression in the training set for further analysis.

**Figure 1:**
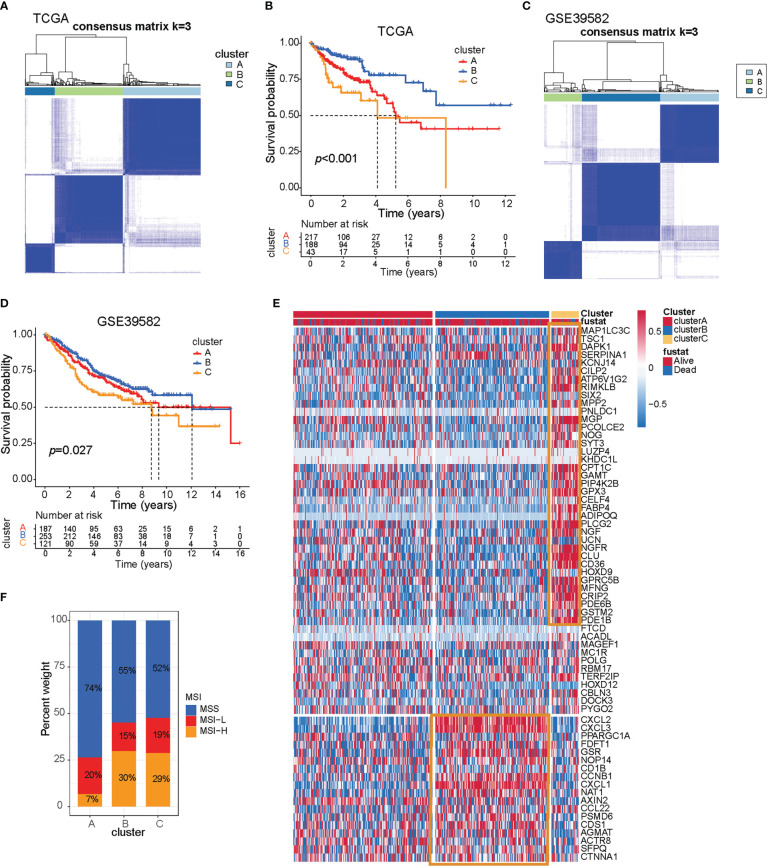
Three prognostic molecular subtypes of colon cancer. **(A)** Clustering heat map based on 66 prognosis-related genes in TCGA-COAD cohort. **(B)** Survival curve of TCGA-COAD patients in different clusters. Survival differences were assessed with a log-rank test. **(C)** Clustering heat map based on 66 prognosis-related genes in the GSE39582 cohort. **(D)** Survival curve of GSE39582 patients in different clusters. Survival differences were assessed with a log-rank test. **(E)** Heat map of 66 prognosis-related genes in TCGA-COAD cohort. **(F)** The proportion of microsatellite instable subtypes in A–C subtypes of TCGA-COAD cohort.

Based on these 66 prognostic genes, we attempted to classify COAD patients into different subtypes. The R package, ConsensusClusterPlus, was used to classify patients using unsupervised clustering, resulting in 217 cases in cluster A, 188 cases in cluster B, and 43 cases in cluster C (**Figures 1A, B**). Next, prognostic analysis for the three subtypes revealed a particularly prominent survival advantage in cluster B (**Figure 1B**). To examine the three subtypes, we also used the GEO dataset (GSE39582) to do clustering. As shown in **Figures 1C, D**, we could also get similar results based on the 66 prognostic genes. Interestingly, we found that the prognosis of the three subtypes follows the same trend, with cluster B having better survival than the others (**Figures 1B, D**). This demonstrated that three distinct subtypes did exist in colon cancer.

To explore the survival characteristics of colon cancer, we further examined the characteristics of 66 prognostic genes in different subtypes and found that cluster B was characterized by increased expression of prognostic favorable genes and low expression of adverse prognostic genes. On the contrary, cluster C was characterized by the opposite results of cluster B (**Figure 1E**, yellow box).

Dysfunction of genes in the DNA mismatch repair pathway reduces the ability of cells to repair DNA replication errors and thereby leads to microsatellite-instable (MSI) subtypes of colon cancer (16). The patients with MSI have a higher somatic mutation burden and immune infiltration in the TME compared to their microsatellite-stable (MSS) counterparts (17). Immune checkpoint genes such as CTLA4 and CD274 are more highly expressed in MSI than in MSS patients (18–20). Apart from high sensitivity to immunotherapy, MSI status itself is a good prognostic marker for CRC patients subject to conventional treatment. MSI patients exhibit less clinical aggressiveness and a longer survival time than MSS patients. Further research showed that tumors with MSI subtype were mainly characterized by clusters B and C, while tumors with the MSS subtype were characterized by cluster A, and that also indicated cluster B/C patients may be suitable for receiving immunotherapy (**Figure 1F**).

**Figure 2:**
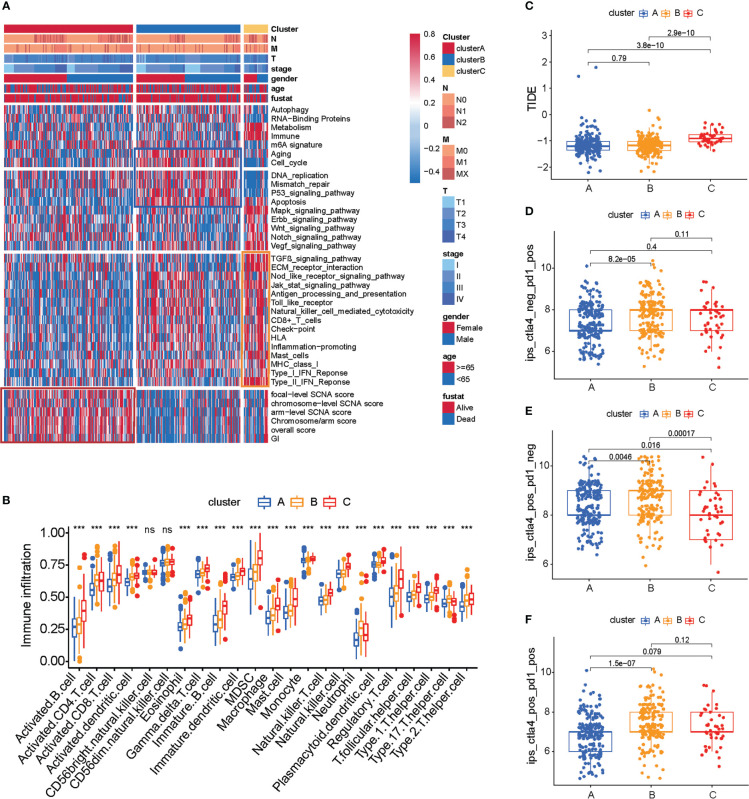
TME cell infiltration characteristics and immunotherapy prediction in distinct three subtypes of colon cancer. **(A)** Heat map of molecular characteristics in three clusters in TCGA-COAD cohort. The four regions (rows) of the heat map respectively represent key tumor processes, tumor-related pathways, immune-related processes, chromosome stability, and somatic cell copy number. **(B)** Analysis of immune cell content in TCGA COAD cohort. The one-way ANOVA test was used for comparison between different groups. ***p < 0.001, ns *p* > 0.05. **(C)** Heat map of hot or cold tumor marker genes in TCGA-COAD cohort. **(D–F)** The relationship between immunotherapy-related scores and patient subtypes in TCGA-COAD cohort. The Wilcoxon test was used for comparison between different subtypes.

The subtypes based on the 66 prognosis genes were significantly sociated with various clinicopathological parameters; cluster C was enriched for T3 tumors and high-grade tumors (**Figure 2A**). Analysis of the biological processes associated with distinct subtypes revealed important patterns. Cluster B was associated with cell cycle, DNA replication, mismatch repair, P53 signaling pathway, and apoptosis. Post-translational modifications of the p53 signaling pathway play an important role in cell cycle progression and stress-induced apoptosis (21). P53-mediated apoptosis may account for the favorable prognosis of cluster B (**Figure 2A**, blue box). By contrast, cluster C tumors were mostly associated with Mapk, Erbb, Wnt, Notch, and Vegf signaling pathways (**Figure 2A**), and this signaling pathway may play an important role in drug resistance (22–24), which may cause a worse prognosis for cluster C. Additionally, cluster A was intermediate between clusters C and B, which is consistent with the prognosis.

We estimated the presence of immune cells by deconvolution of RNA-Seq data (25). To our surprise, cluster C was prominently related to the immune biological process (**Figure 2A**, yellow box). The results from GSVA analysis revealed that cluster C was remarkedly enriched in stromal and carcinogenic activation pathways such as ECM receptor interaction and TGF beta signaling pathway, and it was also remarkedly rich in immune cell function activation, such as CD8+ T cell, antigen processing and presentation, inflammation-promoting, and IFN response. Previous studies demonstrated that tumors with immune-excluded phenotypes also showed the presence of abundant immune cells, while these immune cells were retained in the stroma surrounding tumor cell nests rather than penetrating their parenchyma (26).

Genomic instability (GI) and somatic copy-number alterations (SCNAs) are important in increasing the adaptive potential of the tumor and have been linked with a poor prognosis (27). The SCNA score is a representation of the level of SCNAs occurring in a tumor. For each tumor, the SCNA score was calculated at three different levels: focal, arm, and chromosome level, and the overall score was calculated from the sum of all three levels (15). We found that cluster A tumors were remarkedly enriched with high SCNA and high GI (**Figure 2A**, red box; **Supplementary Table S4**).

We then used the CIBERSORT method, a deconvolution algorithm using support vector regression for determining the immune cell type in tumors, to compare the component differences of immune cells among the three subtypes of colon cancer. We found that there are significant differences in the composition of TME cell types between the three subtypes of colon cancer (**Figure 2B**), which suggested that the three subtypes have distinct TME infiltrating-cell types of tumors. Based on the above analyses, we were surprised to find that three subtypes of colon cancer had significantly distinct TME cell infiltration characterization. Cluster A was classified as an immune-desert phenotype, characterized by the suppression of immunity. Cluster B was classified as an immune-inflamed phenotype, characterized by adaptive immune cell infiltration and immune activation. Cluster C was classified as an immune-excluded phenotype, characterized by innate immune cell infiltration and stromal activation (**Figure 2B**). Interestingly, we found that an immune-excluded state prejudices the survival of colon cancer patients, while immune-inflamed state is a particularly prominent survival advantage in cluster B. To verify the result in TCGA cohort, we next analyzed the TME cell infiltration in GSE39582 (**Supplementary Figures S2A, B**). Again, consistent with the result in TCGA cohort, the immune-inflamed phenotype (cluster B) is preferred for survival (**Figure 1D**).

The above results showed again that three subtypes of colon cancer have distinct TME landscapes. Predicting the response to immunotherapy based on the characterization of TME cell infiltration is a key procedure for increasing the success of existing immunotherapy and exploiting novel immunotherapeutic strategies (28, 29). Therefore, we further predicted the immunotherapy of three subtypes of colon cancer. We found that cluster B had a lower TIDE score and more response to PD1/CTLA4, which indicated that cluster B was more likely to benefit from the immunotherapy. These results indicated that cluster B is suitable for immunotherapy (**Figures 2C–F**; **Supplementary Tables S5**, **S6**).

### 3.2 Characteristics of three subtypes of colon cancer in tumor somatic mutation

**Figure 3:**
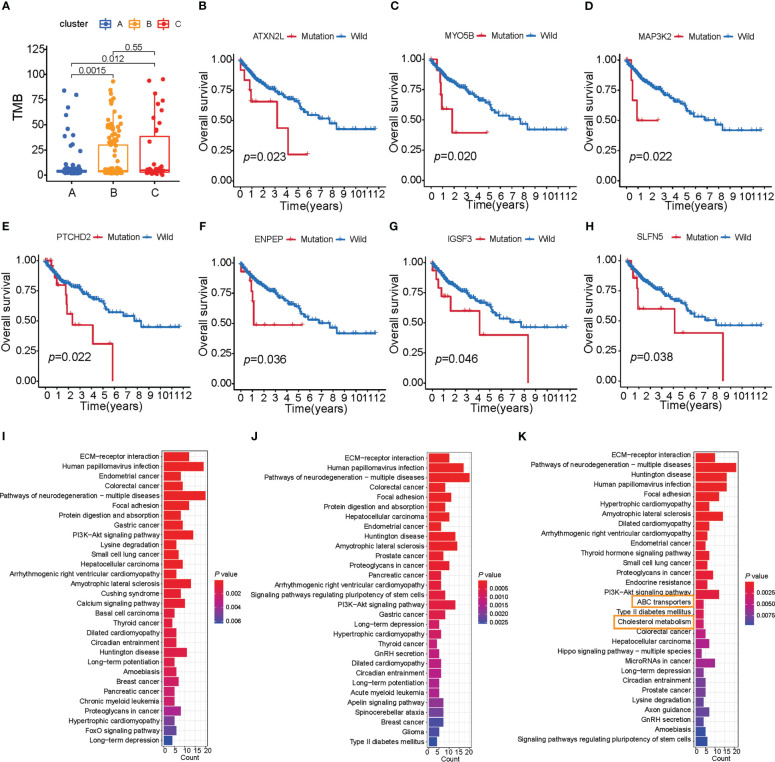
Characteristics of three subtypes of colon cancer in tumor somatic mutation. **(A)** TMB analysis (Wilcoxon test) of patient subtypes in TCGA-COAD cohort. **(B–H)** Survival analysis of key gene mutations (log-rank test). **(I–K)** The signaling pathway of the top 200 mutation genes enriched in three distinct subtypes.

Clinical trials as well as preclinical studies have revealed that patients with high somatic TMB have an enhanced response, long-term survival, and durable clinical benefit when treated with immune checkpoint blockade therapy. We then analyzed the distribution differences of somatic mutation between three subtypes of colon cancer in TCGA-COAD cohort using the maftools package. The TMB quantification analyses confirmed that cluster B was markedly correlated with higher TMB (**Figure 3A**), which confirmed again that cluster B may be more easily responsive to immune checkpoint blockade therapy.

As shown in **Supplementary Figures S3A, B**, cluster A presented a lower tumor mutation burden than clusters B and C, with the average rate of the top 15 mutated genes being 28.3% versus 34.6% and 32.9%, respectively. We also found that cluster A was characterized by a high TP53 mutation, cluster B by TTN mutation, and cluster C by APC mutation (**Supplementary Figures S3D–F**). To further investigate the mutation genes of each subtype of colon cancer, we determined three subtype-related mutations using the maftools package. Given that gene mutation is often related to survival, we analyzed the connection between these mutation genes and survival using the TIMER database (**Supplementary Tables S7**-**S10**). Furthermore, seven genes were found to be related to survival (ATXN2L, IGSF3, MYO5B, PTCHD2, SLFN5, ENPEP, and MAP3K2, *p*< 0.05, **Figures 3B–H**; **Supplementary Table S7**), and all of these seven genes had a high mutation rate in cluster C. These findings indicated that these adverse prognostic gene mutations may also contribute to cluster C’s worse prognosis. Considering that cluster C was characterized by immune activation, we further explore the connection between survival-related gene mutation and CD8+ T-cell infiltration. We found that MAP3K2, ATXN2L, BAZ1B, and PARP14 mutations resulted in high CD8+ T-cell infiltration in tumors (**Supplementary Figures S3G–J**). Our observation above supported our hypothesis that greater TME cell infiltration may result in the worst prognosis for cluster C patients.

Mutational patterns in DNA are derived from mutational processes that result in distinct biological changes occurring during tumorigenesis. Therefore, we examined the pathways in which the mutation gene was enriched in distinct subtypes. We chose a mutation gene that ranks in the top 200 in each of the subtypes, and then ran a KEGG enrichment analysis on these genes. To our surprise, the mutation genes in cluster C were enriched in signaling pathways related to lipid metabolism compared with clusters A and B, such as ABC transporters and cholesterol metabolism (**Figures 3I–K**). Based on studies that show that limiting fatty acid availability can control cancer cell proliferation (30), cluster C patients may have lipid disorders, which may result in a poor survival rate.

### 3.3 Classifier for predicting patient subtypes and drug screening

**Figure 4:**
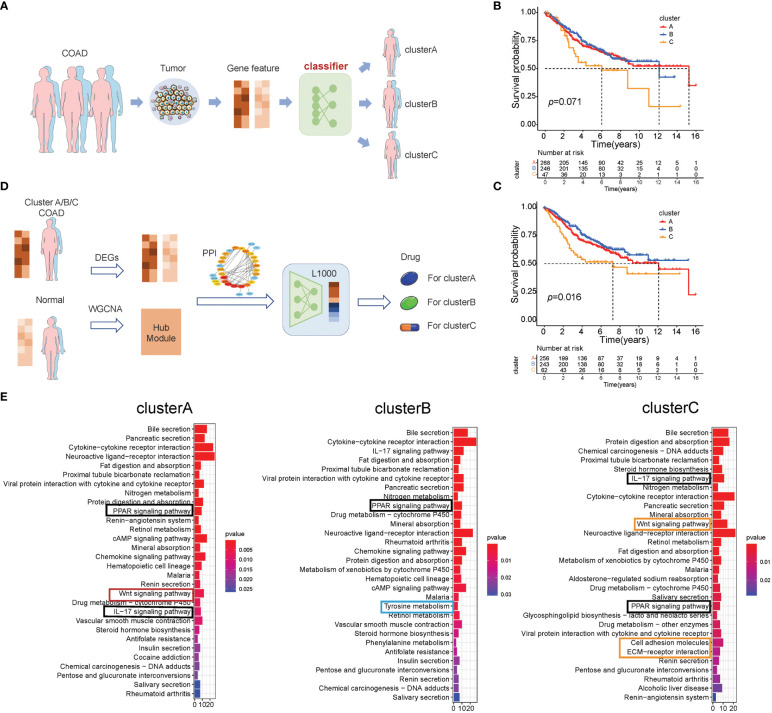
Classification-drug-prediction system. **(A)** Flow chart of the COAD subtype prediction. **(B)** For the GSE39582 cohort, if the distance from the centers of the three subtypes in TCGA-COAD was used as a classification standard, then the result of the survival analysis is not significant (log-rank test). **(C)** For the GSE39582 cohort, the MLP model was used to do classification, and the result of the survival analysis is significant (log-rank test). **(D)** Flow chart of drug screening for different molecular subtypes. **(E)** GO analysis of the intersection genes in the WGCNA and DEGs (patients *vs*. controls) of TCGA-COAD clusters A–C, respectively.

The above results showed that there are three subtypes of colon cancer patients based on the 66 prognostic genes, and cluster B has a prominent survival advantage over cluster A/C, while cluster C has more TME cell infiltration, indicating that these three subtypes have different transcriptome features. Therefore, we hypothesized that patients of different subtypes should be treated differently. Pursuing this, based on the expression of 66 prognostic genes, we compared two different methods, as described in the Methods section. Method (1) was based on unsupervised learning, and method (2), which is an MLP classifier, was based on supervised learning (**Figure 4A**). In cross-validation, the MLP model achieves the highest accuracy (0.919; **Supplementary Table S11**) in the training set when “identity” = “logistic,” “alpha” = 0.01, and “solver” = “lbfgs.” As a result, we used these parameters to set up the MLP model using the whole training set. In the survival curves of the test set (GSE39582), the labels in method (1) cannot distinguish survival states (*p* = 0.071, log-rank test, **Figure 4B**), but the MLP model can distinguish the survival states significantly (*p* = 0.016, log-rank test, **Figure 4C**), which is consistent with the result in the training set (**Figure 1D**). Therefore, this MLP model is used to establish a web app (https://sujiezhulab.shinyapps.io/coad/) that can be used easily by other researchers.

Next, we compared each subtype of colon cancer to normal colon samples and obtained DEGs. We used the WGCNA and PPI to further select hub genes related to each subtype based on these DEGs (**Supplementary Figures S4**-**S6**; **Supplementary Table S12**-**S14**), and these hub genes were used as L1000 input data (https://clue.io/), a tool used to screen drugs that can reverse gene expression from a disease state to a healthy state. In addition, these drugs were regarded as effective drugs for the special disease. In our research, drugs with CMap connectivity (tau) score of<−0.9 were selected and included in our recommendation list (**Figure 4D**). Herein, we used up/down gene signatures to obtain a drug list as adjuvant therapy. Furthermore, we observed that there are some drugs with anti-inflammation effects for cluster B/C, which is consistent with the fact that cluster B/C contain more macrophages (**Supplementary Tables S15**-**S17**).

To further explore the signaling pathways of the DEGs in cluster A/B/C, we performed KEGG enrichment analysis (**Figure 4E**) and found that cluster A/B/C was enriched in some similar signaling pathways, such as the IL-17 signaling pathway and the PPAR signaling pathway. There are also some cluster-specific enriched signaling pathways related to immune and lipid metabolism. Cluster A was associated with the Wnt signaling pathway; cluster B was enriched in tyrosine metabolism; and cluster C was characterized by inflammation signaling pathways such as cell adhesion and ECM–receptor interaction, which supports the results that the drugs for cluster C were anti-inflammatory. Notably, cluster C was also enriched in the Wnt signaling pathway, which indicated that cluster C has a double poorer prognosis feature (**Figure 4E**). Recalling our observations of drugs for the distinct cluster, drugs related to lipid metabolism all existed in three subtypes, which is consistent with our results that the PPAR signaling pathway was enriched in all subtypes.

### 3.4 Construction of MKPC score for prognostic classification of colon cancer patients

Given our observation of distinct prognosis of three subtypes based on 66 prognostic genes, it was notable that the expression of 66 prognostic genes differed significantly among the three subtypes. Therefore, we explored the ability to distinguish prognosis based on 66 prognostic genes in Pan-cancer. To our surprise, when the cancers were divided into three subtypes, there were 15 kinds of cancer showing distinct prognoses, which indicated that these genes were important for cancer patients’ survival (*p<* 0.05, Wilcoxon test, **Supplementary Figures S7A–O**).

To establish a robust prognostic model and avoid the batch effect, the 66 prognostic genes were used for gene pairing, yielding a total of 2,145 (66 * 65/2) gene pairs, 357 of which have a frequency of “gene A > gene B expression” between 20% and 80% in the training set and are considered to have sufficient information to predict survival state. We then obtained 22 gene pairs using univariate Cox regression (**Supplementary Table S18**). In multivariate penalized LASSO regression, 13 gene pairs were selected for survival prediction in the training set. Finally, using multivariate Cox regression, eight gene pairs were identified as being associated with survival difference (**Supplementary Table S16**), and these formed the MKPC score; these eight gene pairs included six risk factors (HR >1) and two protective factors (HR< 1) (**Supplementary Figure S8**; **Supplementary Table S19**). Therefore, the MKPC score is calculated as follows:

Sum = 1.028064 * MPP2|CPT1C − 1.09876 * PPARGC1A|CD36 + 0.573201 * NOG|CD1B + 0.661607 * GAMT|CCL22 − 0.57286 * GSR|MAGEF1 + 0.593767 * NGF|CD1B + 0.927301 * CRIP2|ACTR8

MKPCscore = *e* ^sum^

**Figure 5:**
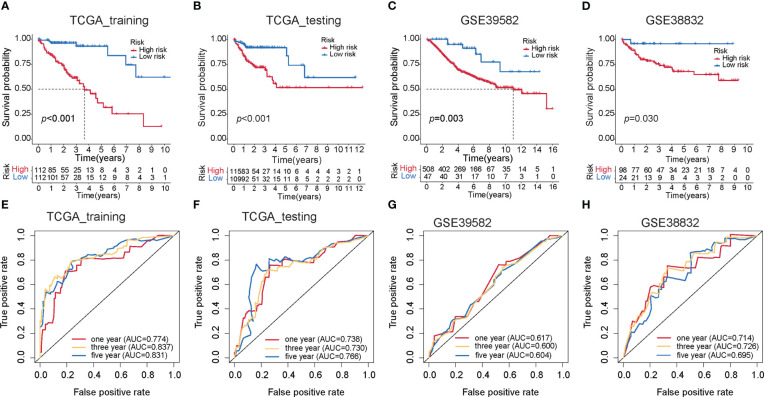
Construction of the MKPC score. Survival curves (log-rank test) of MKPC score in **(A)** TCGA training set, **(B)** TCGA test set, **(C)** GSE39582, **(D)** GSE38832, **(E–H)** ROC curves of MKPC score in **(E)** TCGA training set, **(F)** TCGA test set, **(G)** GSE39582, and **(H)** GSE38832.

To investigate the prognostic performance of the MKPC score, we tested it in four colon cancer patient cohorts (three external test sets and one internal test set). We used the median value of the MKPC score in the training set (1.0544) as the cutoff to separate the high- and low-risk groups in these test cohorts. Notably, the MKPC score showed a wide prognostic value in distinguishing the survival status of colon cancer patients across all cohorts (**Figures 5A–H**), despite differences in patient characteristics and transcriptomic platforms. Consistently, the high-risk group had a worse prognosis in all cohorts (**Figures 5A–D**). These results suggest that the paired MKPC score is a robust prognostic factor in colon cancer. Interestingly, we tested the model in Pan-cancer and observed that the MKPC score has a good performance in distinguishing the survival status of the READ (**Supplementary Figures S8D, E**), which indicated that the MKPC score was related to bowel cancer.

To make the MKPC score more easily usable by other researchers, we built an easy-to-use nomogram based on the MKPC score (**Supplementary Figure S9**). We used the GSE39582 dataset for nomogram construction and validation, which contains various types of clinical information. The GSE39582 cohort was divided into two parts: training set (*n* = 258) and test set (*n* = 137). The training set was used for independent prognostic analysis. We further used the training set to establish the nomogram among the independent risk factors and used AUC-ROCs to verify its performance in the test set. To make this nomogram available to other researchers, including those without programming skills, it was deployed on the server using the “shiny” package in R (https://sujiezhulab.shinyapps.io/coad/).

### 3.5 The clinical and transcriptome characteristics of high- and low-risk patients

**Figure 6:**
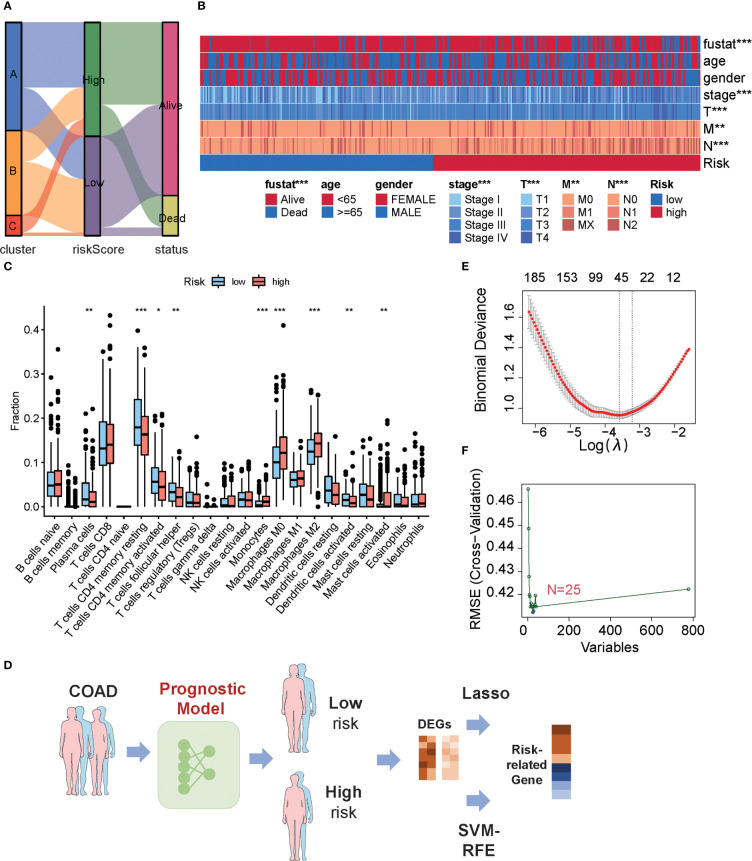
Characteristics of low- or high-risk colon cancer patients. **(A)** The relationship among cluster label, MKPC score, and survival state in TCGA-COAD cohort. **(B)** The difference of clinical features in TCGA-COAD cohort in the MKPC risk group by Chi-square test. ^*^ *p <* 0.05; ^**^ *p <* 0.01; ^***^ *p <* 0.001. **(C)** ssGSEA of immune cell content between different MKPC risk groups in TCGA-COAD cohort (Wilcoxon test). **(D)** Prognosis-related key target analysis process. **(E)** Feature screening based on LASSO regression. **(F)** Feature screening based on Random Forest regression. The blue point corresponds to the smallest RMSE value.

An alluvial diagram was used to visualize the connection between the MKPC score and the three subtypes of colon cancer based on the 66 prognostic genes. We found that most cluster C patients are high-risk, whereas most cluster B patients are low-risk (**Figure 6A**). In clinical practice, patient clinical features, such as age, gender, TNM status, and stage serve as a guide for treating colon cancer. So we looked at how these clinical characteristics differed between the high- and low-risk groups. The higher the grade of the patient, the higher the risk and the lower the chance of survival (**Figure 6B**). Given our results that three subtypes have distinct TME cell infiltration, we observed that the low-risk group had more immune-activated cells and the high-risk group had more immunosuppressive cells (**Figure 6C**). To investigate the potential biological behavior of different risk groups, we performed a GSEA analysis. The high-risk group was markedly enriched in stromal and carcinogenic activation pathways such as ECM receptor interaction, cell adhesion, and MAPK signaling pathways, which is consistent with cluster C, whereas the low-risk group presented enrichment in immune activation pathways such as Natural killer cell-mediated cytotoxicity, JAK-STAT signaling pathway, and Toll-like receptor signaling pathway (**Supplementary Figure S10**).

To obtain risk-related genes, we used the limma packages to obtain DEGs between high- and low-risk groups, and these genes were further selected for risk-related genes (RRGs) using Lasso regression and random forest (**Figures 6D–F**; **Supplementary Table S20**). In addition, the intersected genes between LASSO regression and random forest were regarded as final risk-related genes, yielding 11 genes (**Supplementary Table S20**).

### 3.6 The experiment of risk-related genes

**Figure 7:**
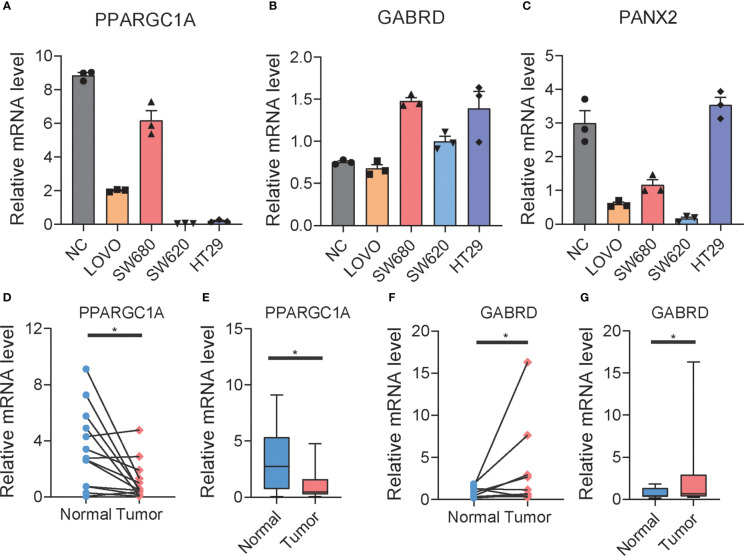
The expression of RRGs in colon cancer. **(A–C)** The expression of PPARGC1A, GABRD, and PANX2 in normal colon cell lines and differential colon cancer cell lines. **(D–G)** The expression of PPARGC1A and GABRD in normal colon tissue and tumor colon tissue of patients. *t*-test was used to compare the expression of genes between normal and tumor. ^*^ *p <* 0.05.

To explore potential colon cancer risk-related genes, we compared the expression of 11 RRGs in normal and tumor tissues (**Supplementary Figures S11A–K**) and found that PANX2 and GABRD are highly expressed in tumors, while PPARGC1A is less expressed in tumors. This difference was consistent with the difference between the low- and high-risk groups. To verify whether these three genes were related to the progression of colon cancer, we used qPCR to examine the expression of those genes in one normal colon cell line and four colon cancer cell lines (**Figures 7A–C**). Among them, PPARGC1A was expressed at a lower level in colon cancer, which is consistent with our previous result that PPARGC1A was expressed at a lower level in a high-risk group, whereas GABRD showed the opposite trend of expression to PPARGC1A in these cell lines, and PANX2 exhibited large expression differences between cell lines. Considering the PPARGC1A and GARBD were consistent in the “tumor *vs*. normal” and “high-risk and low-risk group,” we further explored the expression of these two genes in colon cancer patients. There was a lower expression of PPARGC1A and a high expression of GARBD in colon cancer patients (**Figures 7D–G**). Previous study showed that PGC1-α (PPARGC1A) suppressed melanoma metastasis, and that high PGC1α expression is associated with worse prognosis in metastatic melanomas (31, 32), and that high GARBD expression is associated with poor survival (33). These results indicated that PPARGC1A and GARBD could be potential targets for colon cancer.

## 4 Discussion

An analysis of the molecular basis of inter-patient heterogeneity is a critical first step in understanding why some patients benefit from specific treatments while others fail to benefit. The molecular subtypes of colon cancer can help guide us with individualized treatment. In this study, our results suggest that three distinct three subtypes are based on the expression of 66 prognostic genes from multiple signaling pathways characterized by diverse prognoses, enabling validation in independent cohorts. Integrating RNA subtype classification, pathway information, clinical signatures, immune infiltrate analyses, and TMB status leads us to find that the model of mRNA-based expression subtypes may be associated with a unique response to therapies. Interestingly, cluster B/C patients were characterized by higher immune infiltration and MSI status, especially cluster B with a lower TIDE score, which is the candidate for immunotherapy, while cluster A patients were characterized by the suppression of immunity and higher MSS status, indicating cluster A is not suitable for immunotherapy.

We also observed that the three subtypes of colon cancer had distinct TMB statuses and transcriptome expressions, implying that each subtype of patient should be treated in a unique way. Therefore, we used the L1000 platform to predict the drug for these patients. To better assist clinicians with medication, we developed a classifier that can identify which subtypes of colon cancer a patient has. As a result, patients can be treated based on the expression of their unique genes.

Furthermore, using the novel gene pairing approach, we established a new MKPC score. To the best of our knowledge, this is the first COAD prognostic model that considers multiple signaling pathways at the same time. Using three independent COAD cohorts, we have demonstrated that our MKPC score leads to robust and accurate performance, and that the MKCP score is particularly effective in READ. Our web-tool implementation of the MKPC score and nomogram promotes an easy use of the risk score for COAD.

In short, we summarized the differences between the distinct three subtypes of colon cancer from a comprehensive and multi-omics perspective. At the same time, we developed a classification-drug-prognosis-prediction system that can be used to help clinicians in identifying the best drug for a colon cancer patient. The web tool for predicting patient survival also had a great performance in assisting clinicians. However, the current work has some limitations and areas that could be improved in the future. Cancer, for example, is a molecularly heterogeneous disease whose development has been linked to multiple signaling pathways rather than single pathway genes. Our findings provided novel ideas for identifying the subtypes of colon cancer, which can also be used to distinguish subtypes of other cancers; however, the role of these genes in Pan-cancer needs to be further explored to find a new cancer target. Additionally, the drug lists for cluster A/B/C obtained by L1000 need to be verified with more experiments, although the drugs for cluster A/B/C were consistent with the enriched signaling pathway that the gene features of these three clusters share to some degree.

## Data availability statement

The datasets presented in this study can be found in online repositories. The names of the repository/repositories and accession number(s) can be found within the article/**Supplementary Material**.

## Author contributions

JZ: conceptualization, formal analysis, methodology, software, and writing—original draft. WK: conceptualization, formal analysis, methodology, software, and writing—original draft. LH: formal analysis. SB: investigation. XJ: supervision and writing—review and editing. SZ: supervision, funding acquisition, writing—review and editing, and project administration. All authors contributed to the article and approved the submitted version.

## Supplementary material

Supplementary Figure 1 Flow chart of this study.

Supplementary Figure 2 The characteristics of three subtypes of colon cancer in GSE39582. **(A)** Heat map of 66 prognostic-related genes in GSE39582. **(B)** Immune cell infiltration among three subtypes of colon cancer in GSE39582. One-way ANOVA was used to analysis the difference of immune cell infiltration among three cluster. “*” means *p*<0.05; “**” means *p*<0.01; “***” means *p*<0.001.

Supplementary Figure 3 Mutation analysis of different subtypes in TCGA-COAD cohort. **(A)** Waterfall chart of mutations in Cluster A. **(B)** Word Cloud Analysis of mutations in Cluster A. The character size reflects the number of mutations. **(C)** Waterfall chart of mutations in Cluster B. **(D)** Word Cloud Analysis of mutations in Cluster B. **(E)** Waterfall chart of mutations in Cluster C. **(F)** Word Cloud Analysis of mutations in Cluster C. **(G–J)** The relationship between CD8+ T cell content and gene mutation (Wilcoxon test).

Supplementary Figure 4 The gene signature of cluster A. **(A)** The intersection of DEGs (patients *vs* controls) and WGCNA genes in TCGA-COAD Cluster A patients. **(B)** PPI network of intersection genes.

Supplementary Figure 5 The gene signature of cluster B. **(A)** The intersection of DEGs (patients *vs* controls) and WGCNA genes in TCGA-COAD Cluster B patients. **(B)** PPI network of intersection genes.

Supplementary Figure 6 The gene signature of cluster C. **(A)** The intersection of DEGs (patients *vs* controls) and WGCNA genes in TCGA-COAD Cluster C patients. **(B)** PPI network of intersection genes.

Supplementary Figure 7 Prognostic molecular subtypes of Pan cancer landscape based on the expression of 66 genes. In pan-cancer, the survival curves of 15 cancers whose survival status can be distinguished by 66 prognostic-related genes significantly. Log Rank test is used in this process. ACC, Adrenocortical Carcinoma; BRCA, Breast Invasive Carcinoma; CESC, Cervical Squamous Cell carcinoma and Endocervical Adenocarcinoma; KIRC, Kidney Renal Clear Cell Carcinoma; LGG, Brain Lower Grade Glioma; LIHC, Liver Hepatocellular Carcinoma; LUSC, Lung Squamous Cell Carcinoma; MESO, Mesothelioma; OV, Ovarian Serous Cystadenocarcinoma; READ, Rectum Adenocarcinoma; SARC, Sarcomav; SKCM, Skin Cutaneous Melanoma; THYM, Thymoma; UCEC, Uterine Corpus Endometrial Carcinoma; UVM, Uveal Melanoma.

Supplementary Figure 8 Construction MKPC score. **(A)** The partial likelihood deviance in cross-validation (CV) as a function of the penalty coefficient lambda. The dotted line shows the lambda value of 0.03 at the minimum partial likelihood deviance level, suggesting 13 genes as optimal predictive features. Standard errors are calculated over 1000 CV rounds. **(B)** The coefficients of the 13 genes as a function of the penalty coefficient (lambda). **(C)** The hazard ratios of the MKPC signature genes based on multivariate Cox regression in the training set. The asterisks indicate the statistical significance: **p*<0.05, ***p*<0.01, ****p*<0.001 (Wald’s test). **(D)** Survival curves (Log Rank test) of MKPC score in READ. (E) ROC curves (Log Rank test) of MKPC score in READ.

Supplementary Figure 9
**(A)**The idea of web. **(B)**The nomogram based on MKPC score.

Supplementary Figure 10 GSEA analysis for TCGA-COAD cohort. **(A)** Enriched KEGG pathways in high risk group. **(B)** Enriched KEGG pathways in low risk group.

Supplementary Figure 11 The expression of RRGs in normal colon tissue and colon cancer tissue. **(A–K)** The expression of CCDC151, GAMT, LRFN1, MPP2, NGF, NOG, NXPE1, PANX2, PCOLCE2, PPARGC1A and GABRD in normal colon tissue and colon cancer tissue.

Supplementary Table 1 The summary of clinical information of collected cohorts.

Supplementary Table 2 The collected 183 genes from multiple signaling pathways.

Supplementary Table 3 The significant genes in UniCox regression.

Supplementary Table 4 The SCNA and GI sore of the colon cancer patients.

Supplementary Table 5 The TIDE score of Cluster A/B/C.

Supplementary Table 6 The predicted response to immunotherapy of Cluster A/B/C.

Supplementary Table 7 The different mutations between A and C clusters using Fisher test.

Supplementary Table 8 The different mutations between B and C clusters using Fisher test.

Supplementary Table 9 The intersected mutations between “A *vs* C clusters” and “B *vs* C clusters”.

Supplementary Table 10 The mutations related to survival (Log-Rank test).

Supplementary Table 11 Cross validation of MLP model in TCGA-COAD cohort.

Supplementary Table 12 The gene signature of A cluster.

Supplementary Table 13 The gene signature of B cluster.

Supplementary Table 14 The gene signature of C cluster.

Supplementary Table 15 The recommended drugs of A cluster.

Supplementary Table 16 The recommended drugs of B cluster.

Supplementary Table 17 The recommended drugs of C cluster.

Supplementary Table 18 The UniCox regression of gene-pairs.

Supplementary Table 19 The parameters of MKPC signature (MultiCox regression).

Supplementary Table 20 The risk related genes.
