# Supplementary figures and images for: Identification of immunotherapy and chemotherapy-related molecular subtypes in colon cancer by integrated multi-omics data analysis
Source: Front Immunol. 2023 Mar 20;14:1142609. doi: 10.3389/fimmu.2023.1142609 (PMC10067602; doi:10.3389/fimmu.2023.1142609)

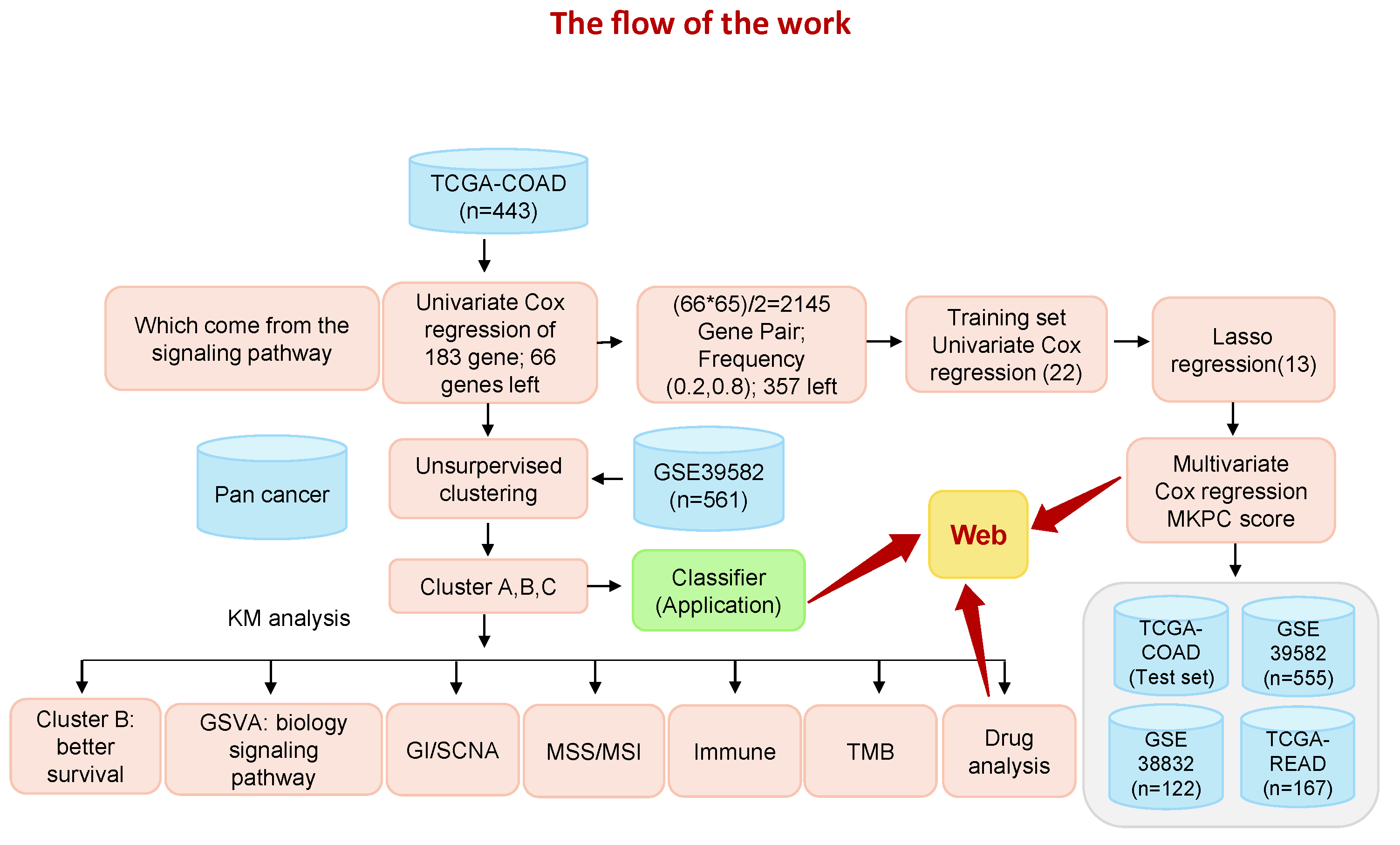

Supplement: Supplementary Figure 1 — Flow chart of this study. [file Image_1.tiff]

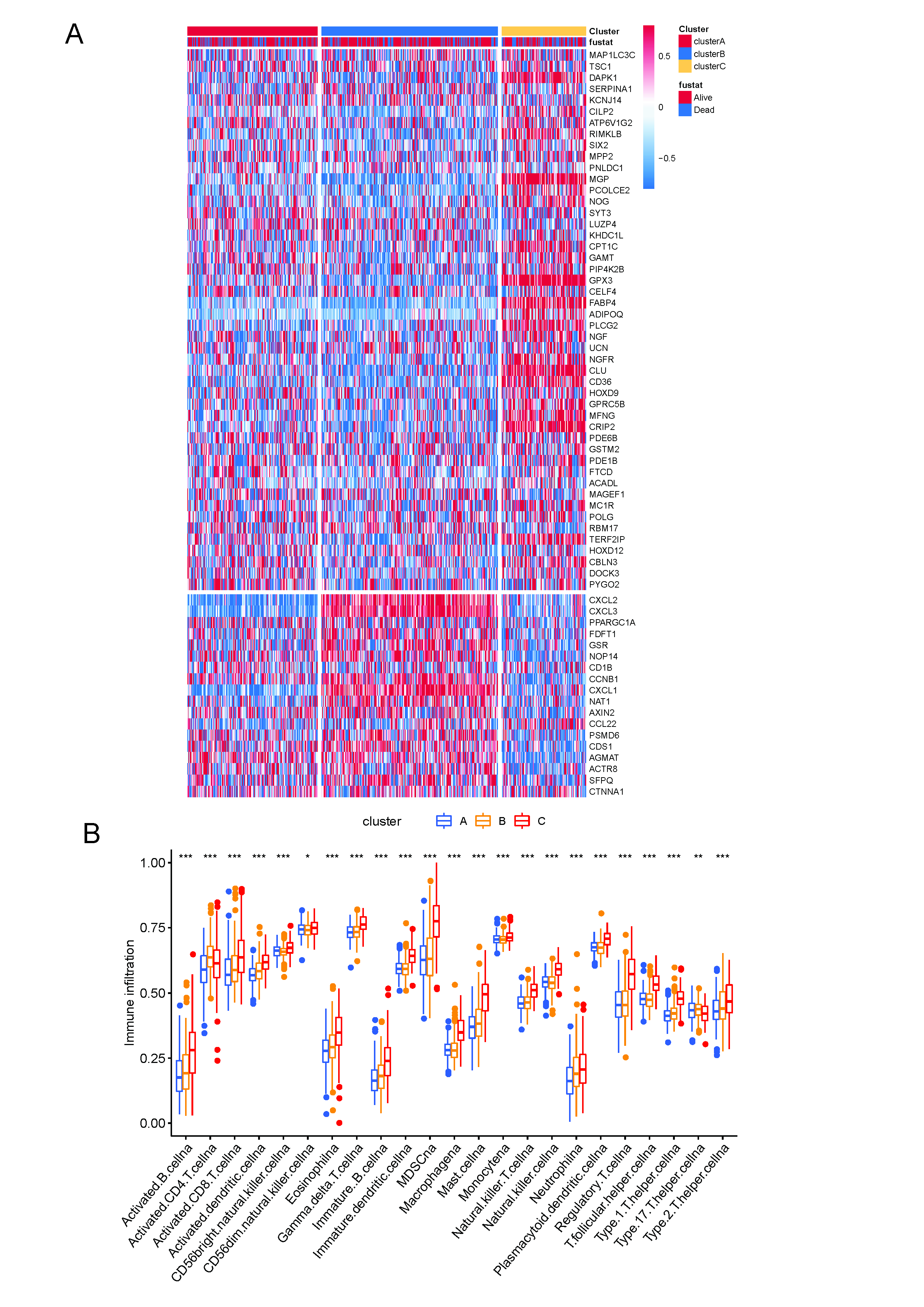

Supplement: Supplementary Figure 2 — The characteristics of three subtypes of colon cancer in GSE39582. (A) Heat map of 66 prognostic-related genes in GSE39582. (B) Immune cell infiltration among three subtypes of colon cancer in GSE39582. One-way ANOVA was used to analysis the difference of immune cell infiltration among three cluster. “*” means p<0.05; “**” means p<0.01; “***” means p<0.001. [file Image_2.tiff]

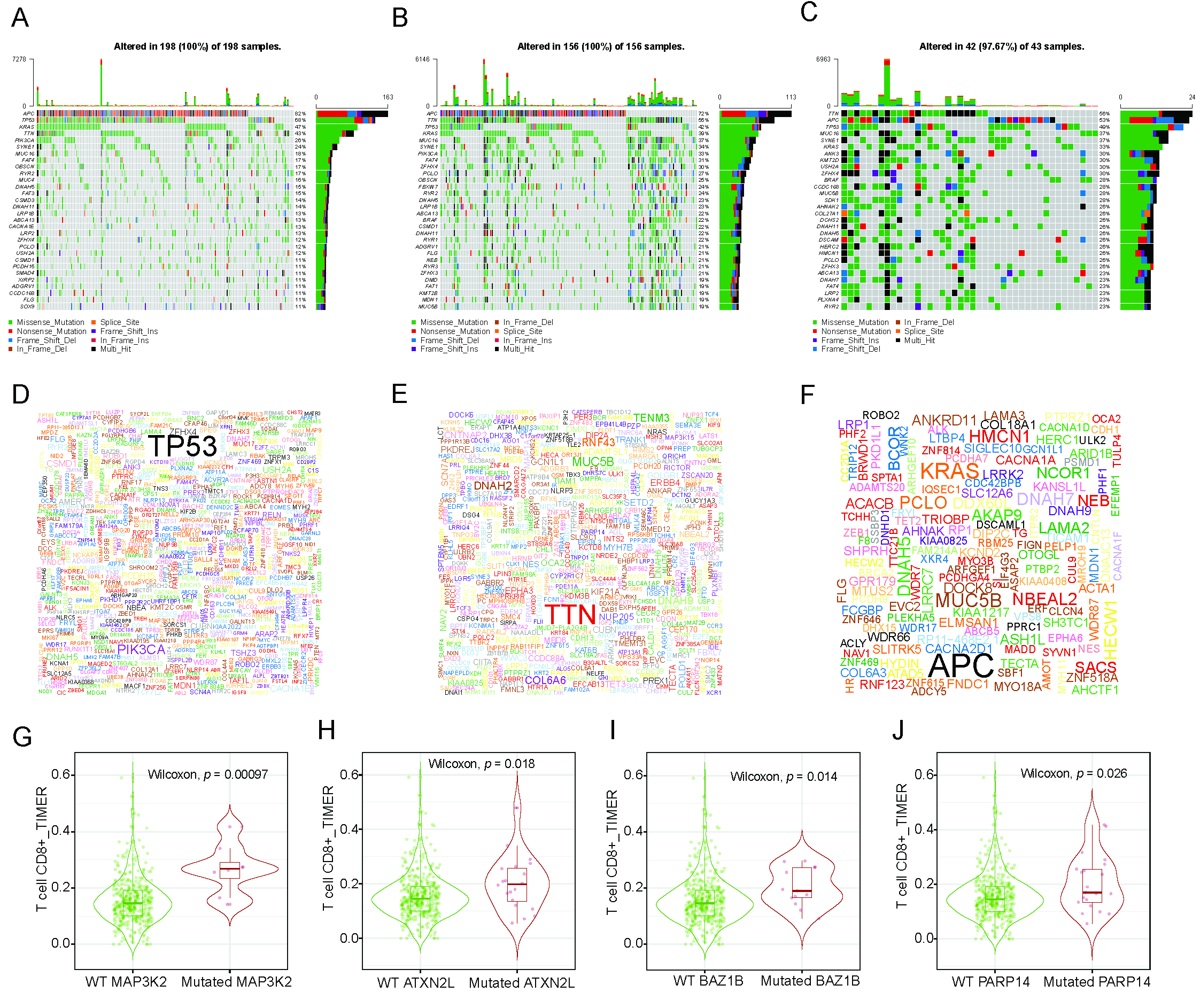

Supplement: Supplementary Figure 3 — Mutation analysis of different subtypes in TCGA-COAD cohort. (A) Waterfall chart of mutations in Cluster A. (B) Word Cloud Analysis of mutations in Cluster A. The character size reflects the number of mutations. (C) Waterfall chart of mutations in Cluster B. (D) Word Cloud Analysis of mutations in Cluster B. (E) Waterfall chart of mutations in Cluster C. (F) Word Cloud Analysis of mutations in Cluster C. (G–J) The relationship between CD8+ T cell content and gene mutation (Wilcoxon test). [file Image_3.tiff]

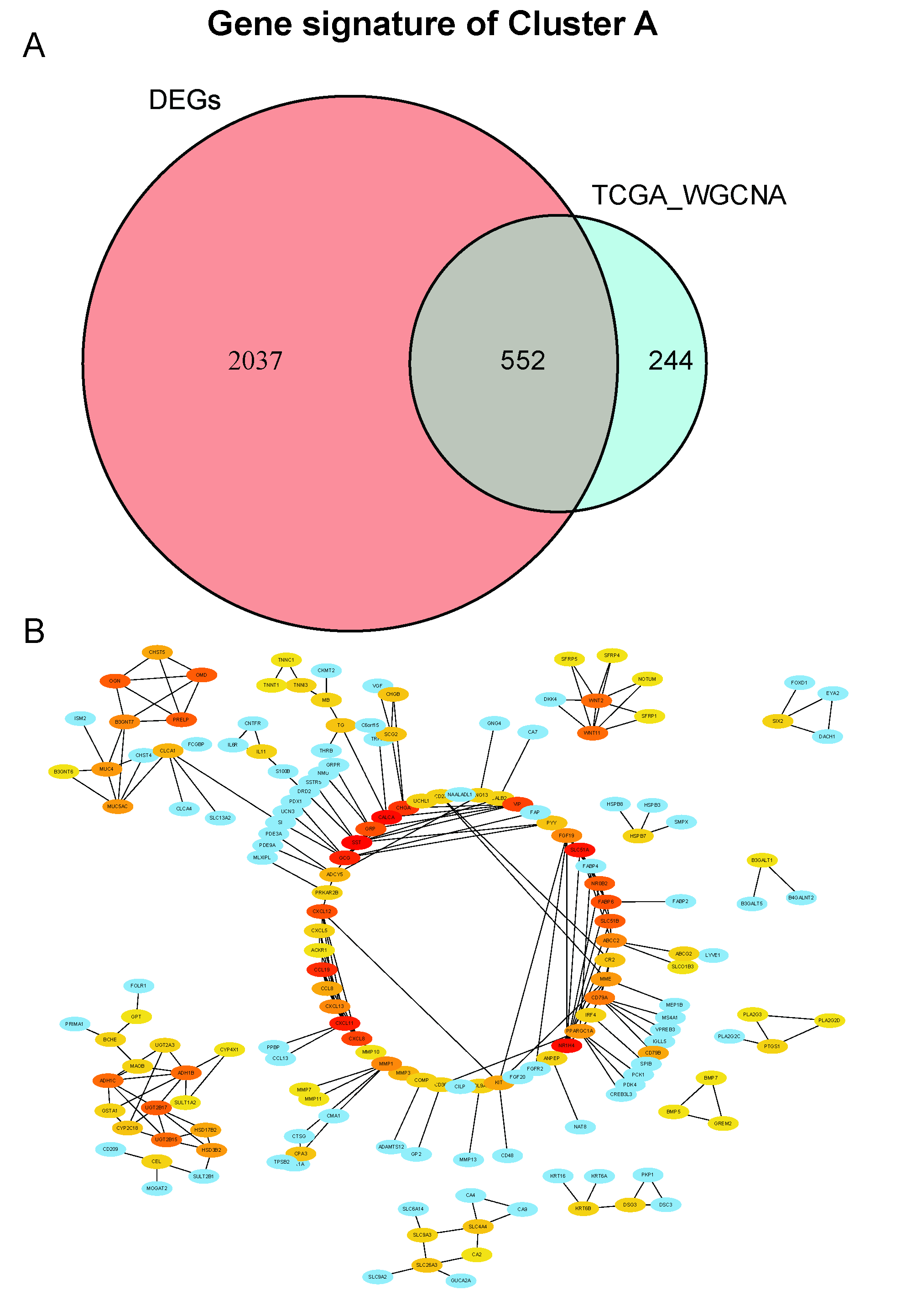

Supplement: Supplementary Figure 4 — The gene signature of cluster A. (A) The intersection of DEGs (patients vs controls) and WGCNA genes in TCGA-COAD Cluster A patients. (B) PPI network of intersection genes. [file Image_4.tiff]

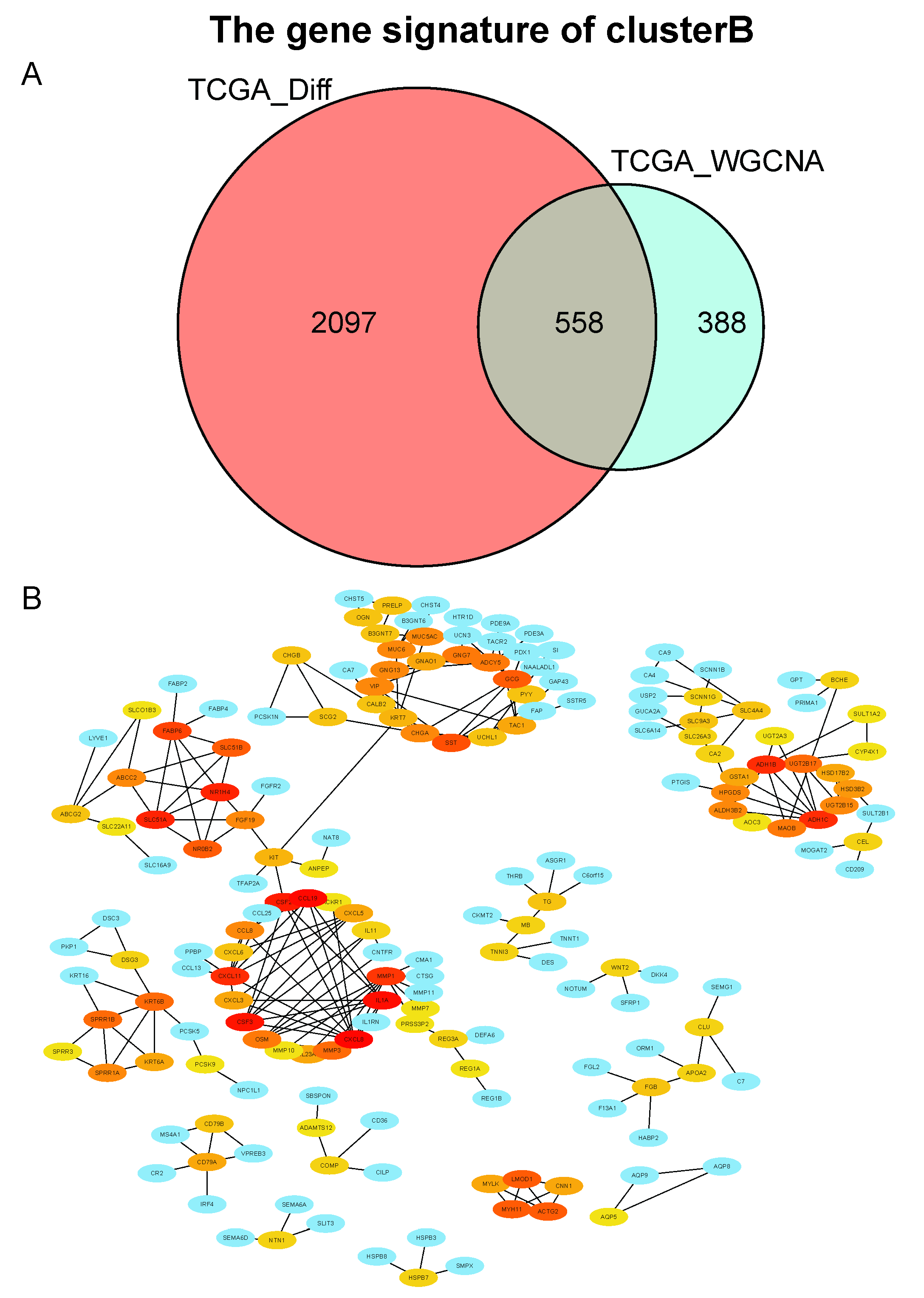

Supplement: Supplementary Figure 5 — The gene signature of cluster B. (A) The intersection of DEGs (patients vs controls) and WGCNA genes in TCGA-COAD Cluster B patients. (B) PPI network of intersection genes. [file Image_5.tiff]

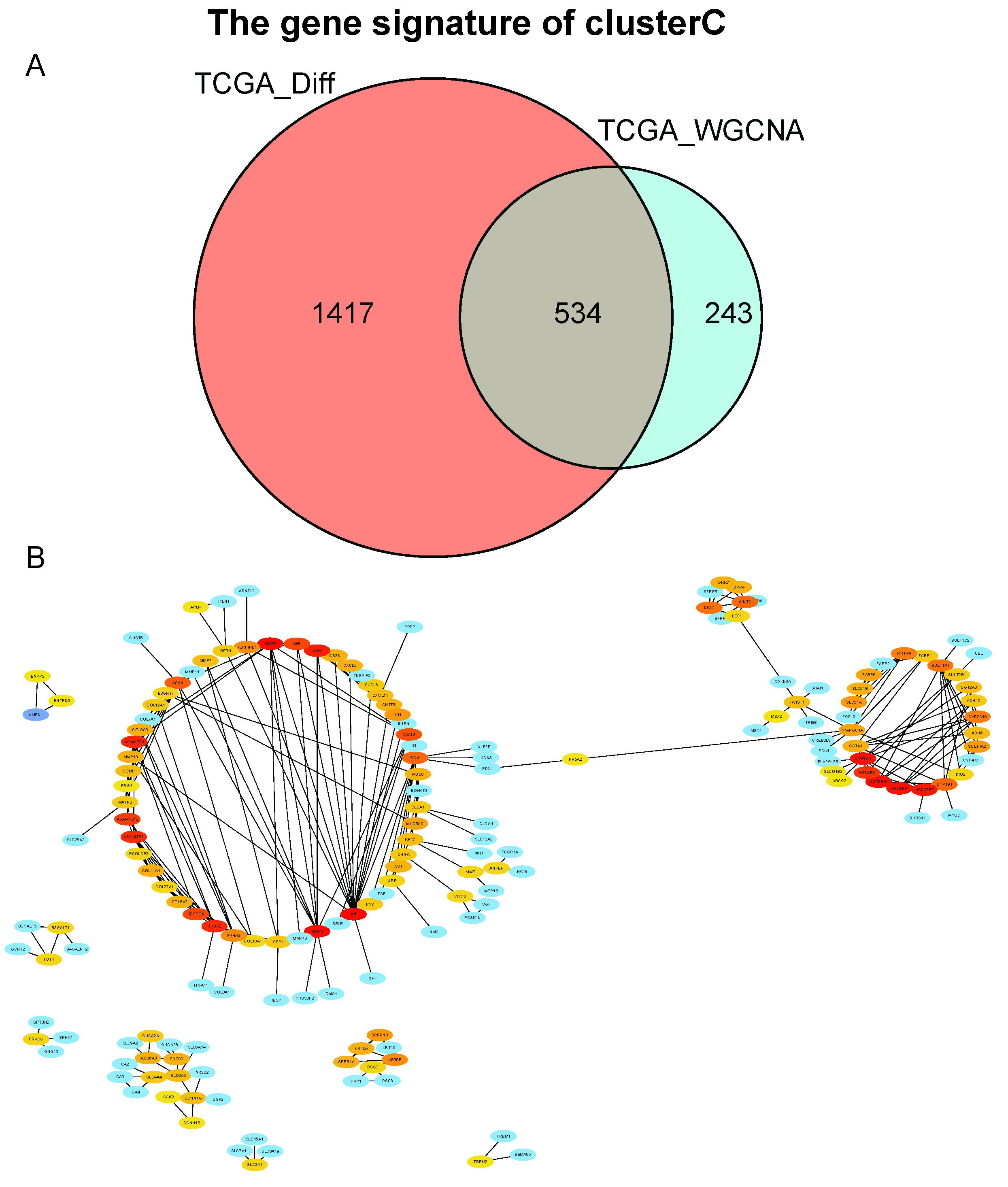

Supplement: Supplementary Figure 6 — The gene signature of cluster C. (A) The intersection of DEGs (patients vs controls) and WGCNA genes in TCGA-COAD Cluster C patients. (B) PPI network of intersection genes. [file Image_6.tiff]

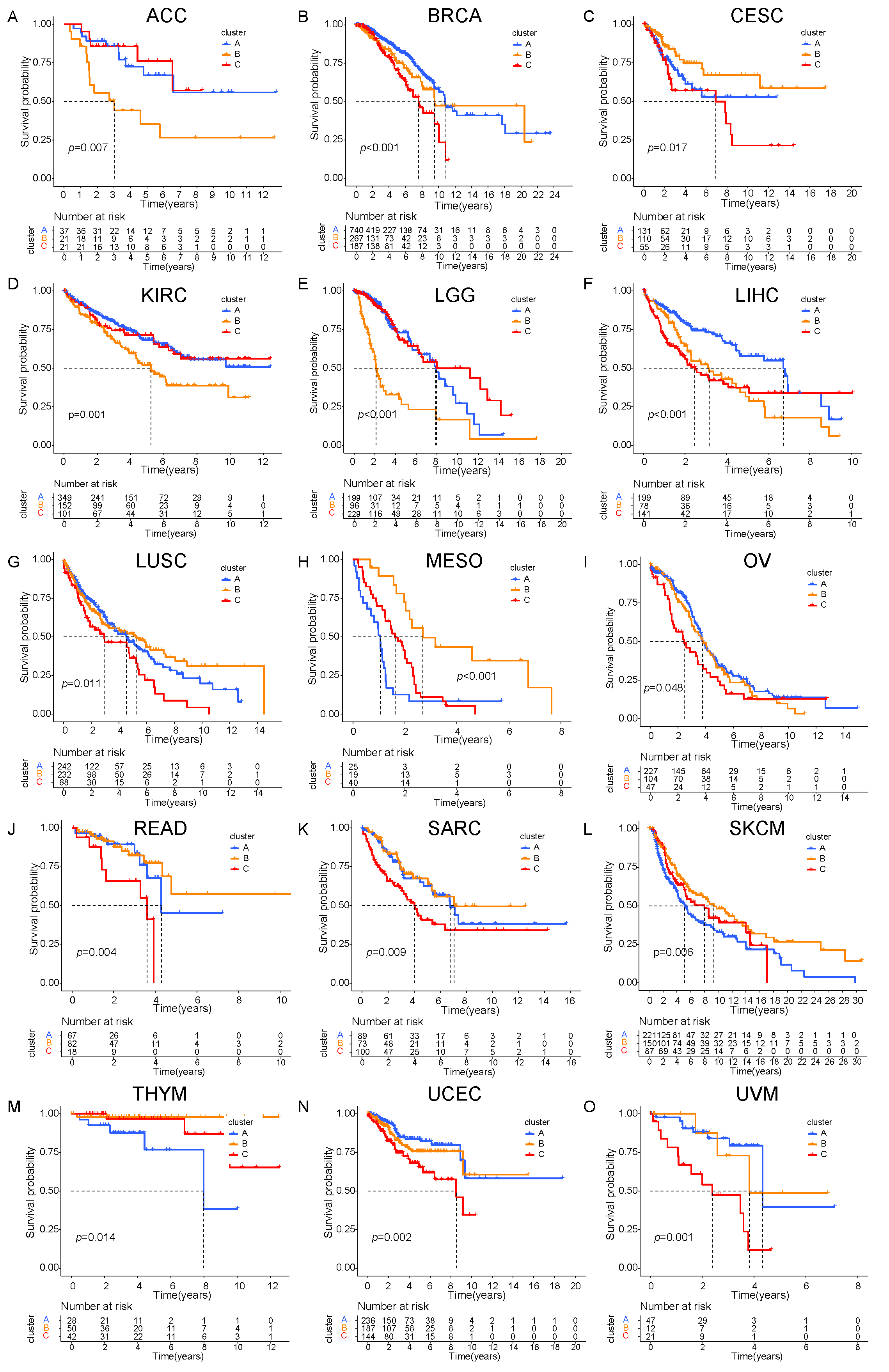

Supplement: Supplementary Figure 7 — Prognostic molecular subtypes of Pan cancer landscape based on the expression of 66 genes. In pan-cancer, the survival curves of 15 cancers whose survival status can be distinguished by 66 prognostic-related genes significantly. Log Rank test is used in this process. ACC, Adrenocortical Carcinoma; BRCA, Breast Invasive Carcinoma; CESC, Cervical Squamous Cell carcinoma and Endocervical Adenocarcinoma; KIRC, Kidney Renal Clear Cell Carcinoma; LGG, Brain Lower Grade Glioma; LIHC, Liver Hepatocellular Carcinoma; LUSC, Lung Squamous Cell Carcinoma; MESO, Mesothelioma; OV, Ovarian Serous Cystadenocarcinoma; READ, Rectum Adenocarcinoma; SARC, Sarcomav; SKCM, Skin Cutaneous Melanoma; THYM, Thymoma; UCEC, Uterine Corpus Endometrial Carcinoma; UVM, Uveal Melanoma. [file Image_7.tiff]

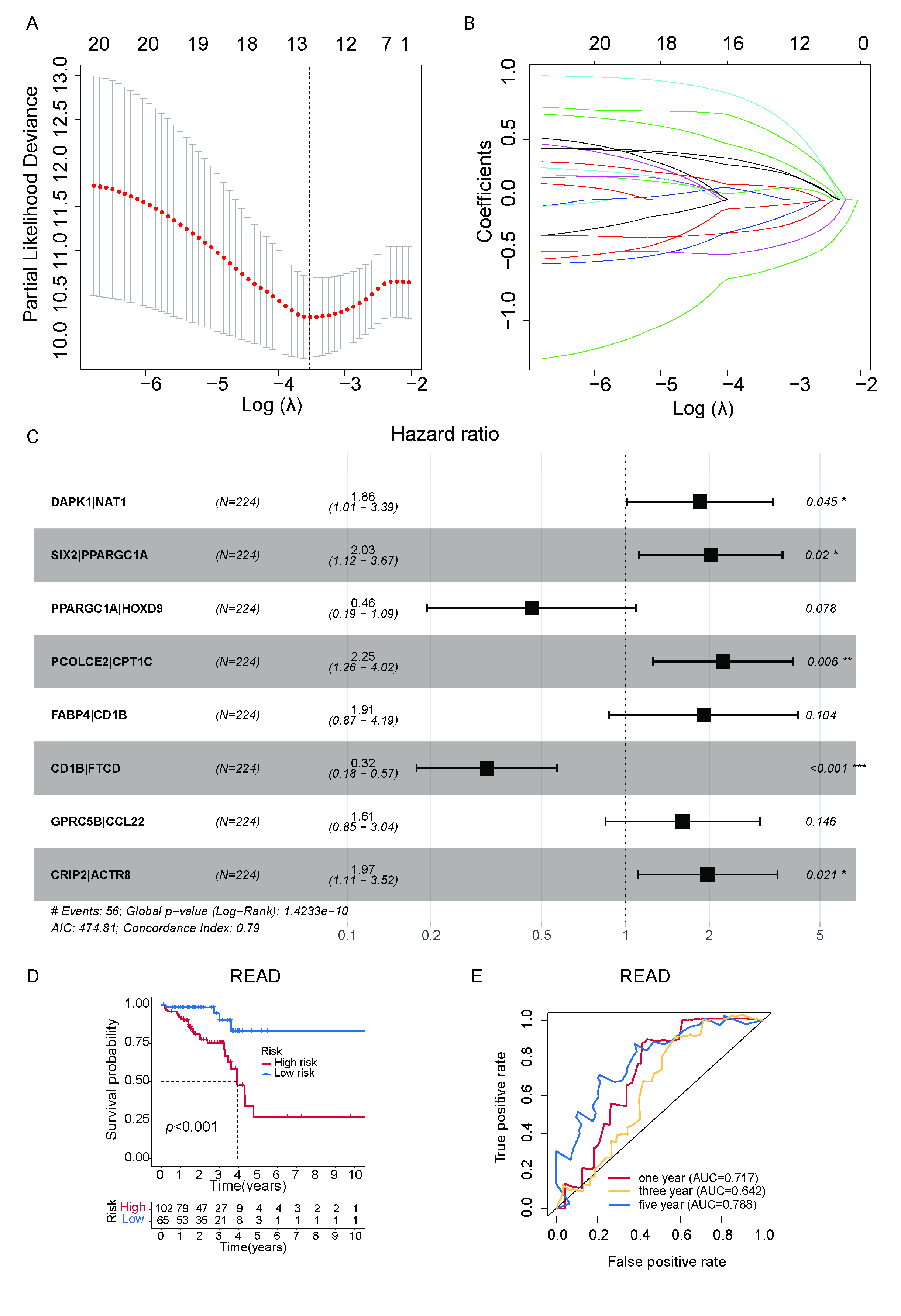

Supplement: Supplementary Figure 8 — Construction MKPC score. (A) The partial likelihood deviance in cross-validation (CV) as a function of the penalty coefficient lambda. The dotted line shows the lambda value of 0.03 at the minimum partial likelihood deviance level, suggesting 13 genes as optimal predictive features. Standard errors are calculated over 1000 CV rounds. (B) The coefficients of the 13 genes as a function of the penalty coefficient (lambda). (C) The hazard ratios of the MKPC signature genes based on multivariate Cox regression in the training set. The asterisks indicate the statistical significance: *p<0.05, **p<0.01, ***p<0.001 (Wald’s test). (D) Survival curves (Log Rank test) of MKPC score in READ. (E) ROC curves (Log Rank test) of MKPC score in READ. [file Image_8.tiff]

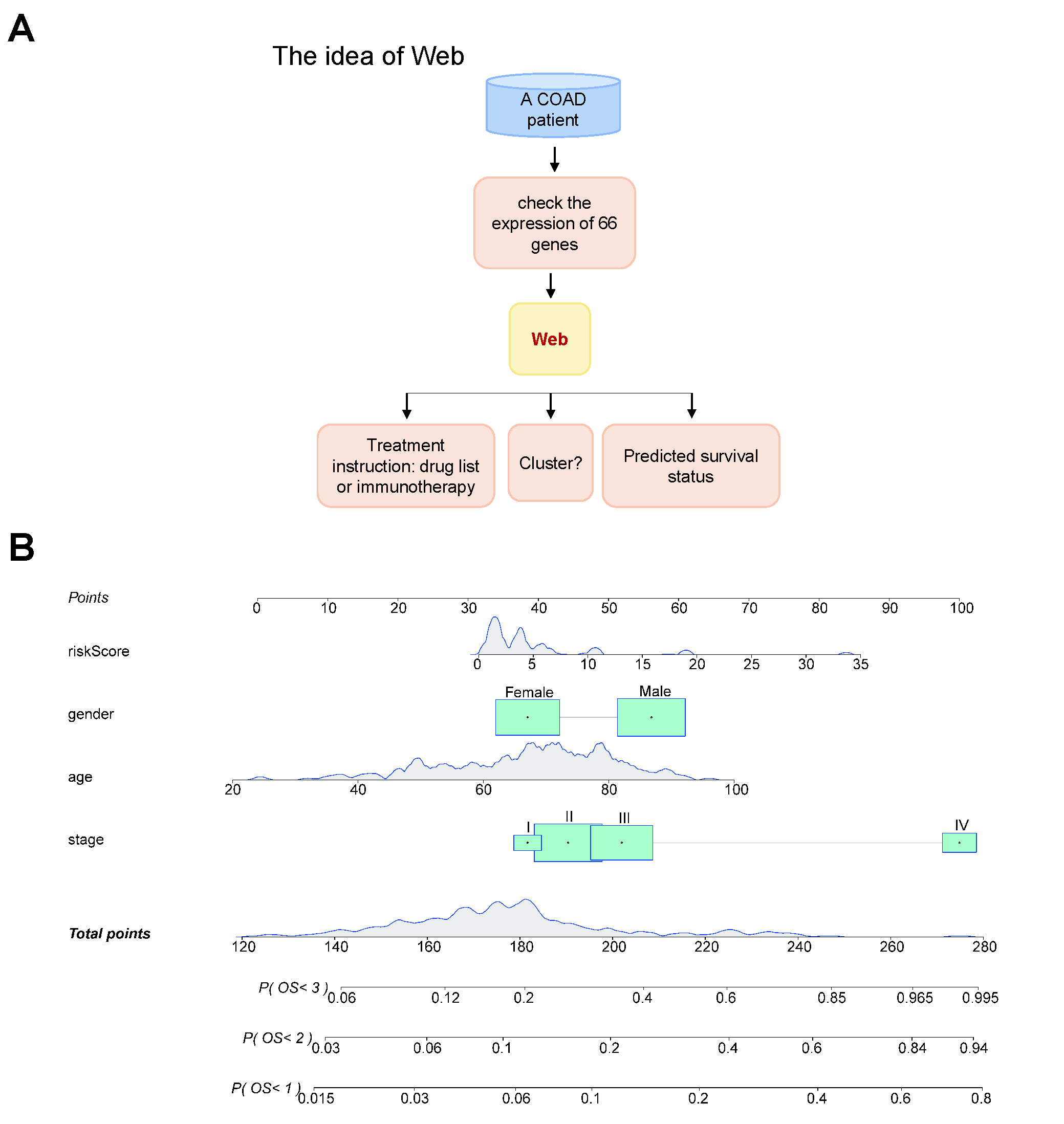

Supplement: Supplementary Figure 9 — (A)The idea of web. (B)The nomogram based on MKPC score. [file Image_9.tiff]

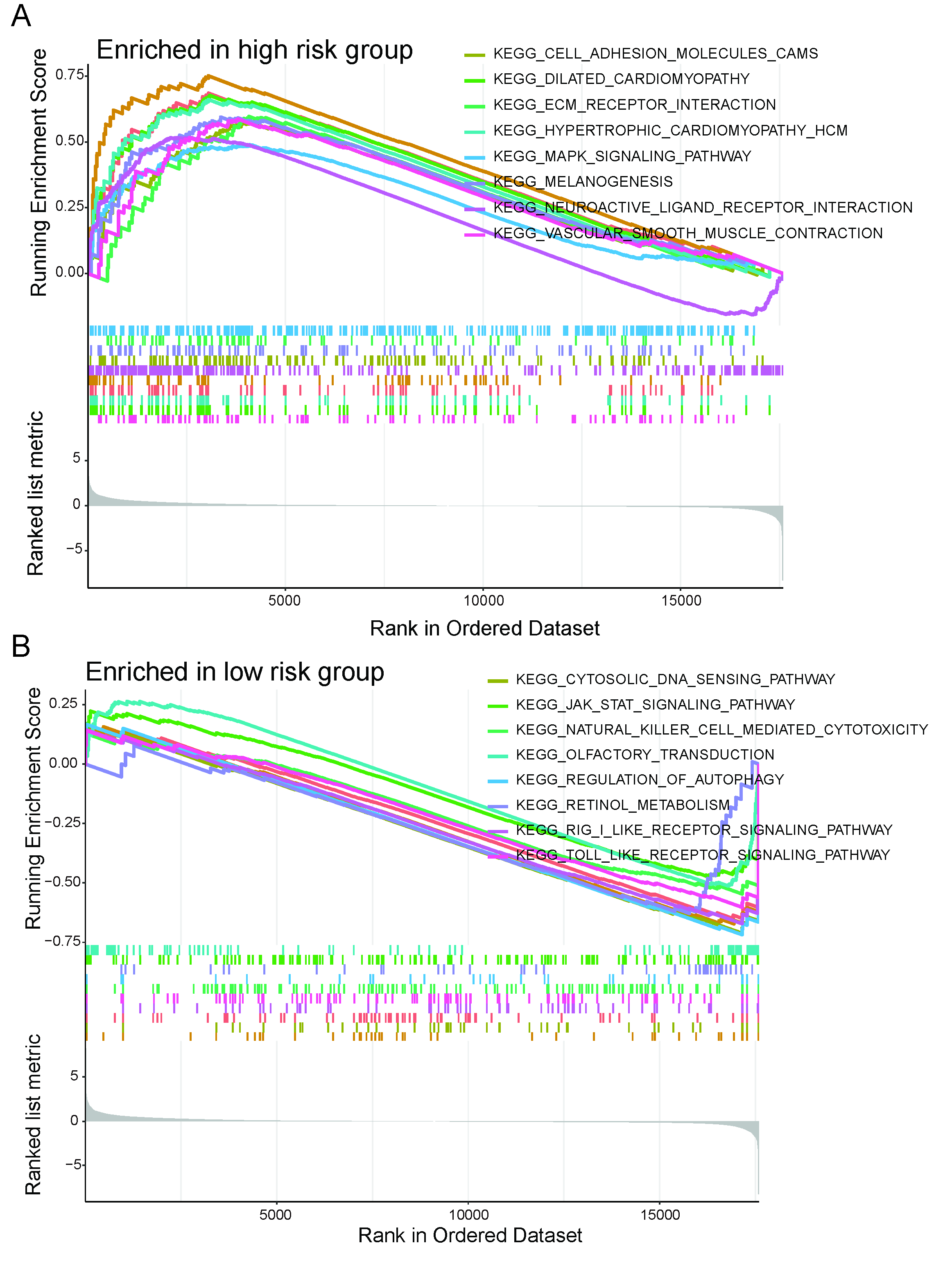

Supplement: Supplementary Figure 10 — GSEA analysis for TCGA-COAD cohort. (A) Enriched KEGG pathways in high risk group. (B) Enriched KEGG pathways in low risk group. [file Image_10.tiff]

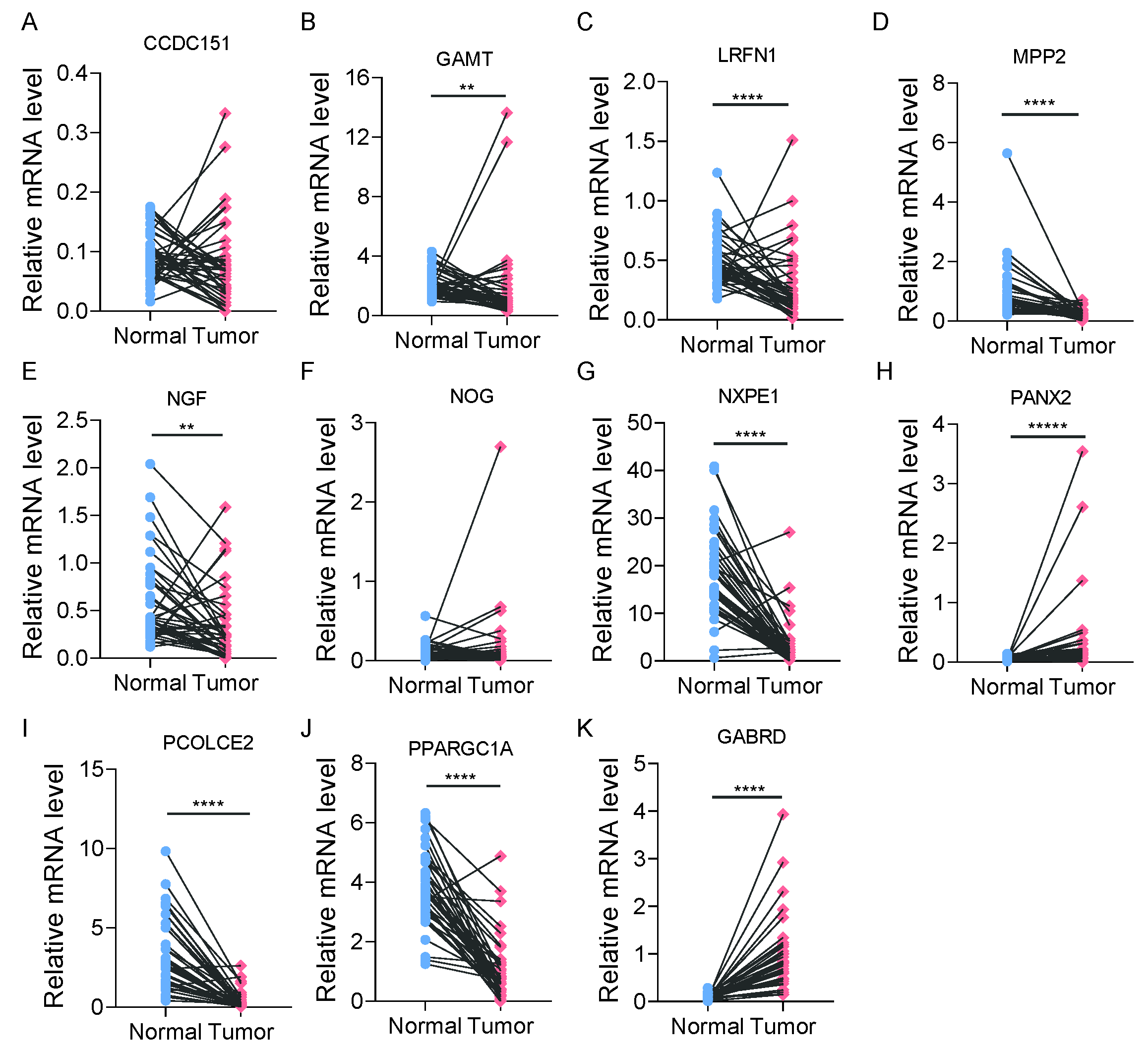

Supplement: Supplementary Figure 11 — The expression of RRGs in normal colon tissue and colon cancer tissue. (A–K) The expression of CCDC151, GAMT, LRFN1, MPP2, NGF, NOG, NXPE1, PANX2, PCOLCE2, PPARGC1A and GABRD in normal colon tissue and colon cancer tissue. [file Image_11.tiff]
